# *In vitro* chondrogenesis of rat adipose-derived stem cells combined with sodium alginate hydrogel scaffold induced by cartilage-derived morphogenetic protein-1

**DOI:** 10.3389/fbioe.2026.1904331

**Published:** 2026-09-11

**Authors:** Changzhi Huang, Nanyi Xu, Lei Zhang, Kaiyuan Zheng, Wenrong Zhang, Shiqin Zhuo, Junli Li, Xiaoyong Wang, Shuqiang Lu, Jiuzao Lin

**Affiliations:** 1 Department of Joint Surgery and Sports Medicine, Ningde Municipal Hospital, Ningde, Fujian, China; 2 Teaching Hospital of Ningde Normal University School of Medicine, Ningde, Fujian, China; 3 Ningde Clinical Medical College of Fujian Medical University, Ningde, Fujian, China; 4 Department of Orthopaedics, Pingnan County General Hospital, Ningde Municipal Hospital Medical Group, Pingnan, Fujian, China

**Keywords:** adipose-derived stem cells, cartilage-derived morphogenetic protein-1, chondrogenic differentiation, sodium alginate hydrogel, three-dimensional culture, tissue engineering

## Abstract

**Objective:**

To explore the feasibility of chondrogenic differentiation of rat adipose-derived stem cells (ADSCs) combined with sodium alginate (NaAlg) hydrogel scaffolds induced by cartilage-derived morphogenetic protein 1 (CDMP-1).

**Methods:**

ADSCs were isolated and cultured *in vitro* from rat inguinal fat pads using type I collagenase digestion, centrifugation and adherent culture methods. Flow cytometry was applied for cell identification, and passage 3 ADSCs were used for subsequent experiments. ① Biocompatibility evaluation of materials: ADSCs were co-cultured with NaAlg hydrogel scaffolds. Cell cytoskeleton staining, cell migration assays and cell proliferation tests were performed to assess the biosafety of the scaffolds. ② *In vitro* chondrogenesis experiment: According to the addition of CDMP-1 and application of three-dimensional NaAlg culture, the cells were divided into four groups: ADSCs group, ADSCs + NaAlg group, CDMP-1 + ADSCs group, and CDMP-1 + ADSCs + NaAlg group. Cell proliferation activity was detected by Cell Counting Kit-8 (CCK-8) at days 1, 3, 5, 7 and 9 after chondrogenic induction. Toluidine blue staining for sulfated glycosaminoglycans (GAGs) and immunohistochemical staining for collagen II (COL-II) were conducted at days 7 and 14 of induction to observe chondrogenic differentiation. Quantitative real-time polymerase chain reaction (qRT-PCR) and immunofluorescence staining were adopted to detect the mRNA and protein expression levels of chondrogenic marker genes *SOX9, COL2A1* and *ACAN* at days 7 and 14 after induction.

**Results:**

Flow cytometry results showed that ADSCs were positive for surface antigens CD29, CD44 and CD105, and negative for CD31 and CD45. After chondrogenic induction, the cell proliferation curves of each group presented an approximate “S” shape, and there were statistically significant differences among all groups (*P* < 0.05). ① Material biosafety and biocompatibility assessment: Phalloidin cytoskeleton staining, scratch assay, Transwell assay, CCK-8 assay and live/dead cell staining verified that NaAlg hydrogel scaffolds exerted no adverse effects on cell morphology, migration capacity or proliferation activity. ② *In vitro* chondrogenesis experiment: Positive results of toluidine blue staining for sulfated GAGs and immunohistochemical staining for COL-II were observed in all groups at days 7 and 14 of induction, and the expression of GAGs and COL-II was the strongest in the CDMP-1 + ADSCs + NaAlg group. At the same time points, the expression levels of chondrogenic markers SOX9, COL-II and ACAN were significantly higher in CDMP-1-treated groups (CDMP-1 + ADSCs group and CDMP-1 + ADSCs + NaAlg group) than those in the control groups (ADSCs group and ADSCs + NaAlg group). Moreover, the expression of SOX9, COL-II and ACAN in NaAlg hydrogel scaffold culture groups (ADSCs + NaAlg group and CDMP-1 + ADSCs + NaAlg group) was remarkably upregulated compared with that in two-dimensional culture groups (ADSCs group and CDMP-1 + ADSCs group).

**Conclusion:**

NaAlg hydrogel scaffold possesses favorable biosafety as a cell-carrier scaffold. CDMP-1 can effectively induce chondrogenic differentiation of ADSCs seeded on NaAlg hydrogel scaffolds *in vitro*. This strategy is expected to provide a novel tissue engineering approach for the repair of cartilage defects.

## 1 Introduction

Articular cartilage is a highly specialized connective tissue primarily composed of chondrocytes and extracellular matrix. It performs critical physiological functions including load bearing, shock absorption and friction reduction within joints. Nevertheless, articular cartilage is devoid of blood vessels, nerves and lymphatic vessels, resulting in an extremely poor intrinsic self-repair capacity after injury. Once cartilage defects or degeneration occur, spontaneous healing is hardly achieved, which may eventually lead to joint dysfunction and severely impair patients’ quality of life. Currently, cartilage damage has become one of the urgent clinical challenges in orthopedics and sports medicine (Yang et al., 2024). Statistics indicate that more than 500 million people worldwide suffer from cartilage injuries and related musculoskeletal disorders (Huh et al., 2026). Conventional therapeutic approaches, such as arthroscopic debridement, microfracture and autologous chondrocyte transplantation, have distinct limitations. Microfracture mainly generates fibrocartilage with inferior biomechanical properties, which tends to degenerate over the long term (Kim et al., 2024). Autologous chondrocyte transplantation is hampered by donor site injury, limited graft sources and mismatched biomechanics, failing to meet the clinical demands for cartilage repair (Tian et al., 2024). Accordingly, exploring efficient and feasible strategies for cartilage repair is of great clinical significance and research value.

The emergence of tissue engineering has brought novel insights into the repair of cartilage defects. Its core principle is to establish a tripartite repair system consisting of seed cells, scaffold materials and inductive factors. By mimicking the physiological microenvironment for cartilage growth, this system enables the regeneration and functional reconstruction of cartilage tissue (Johnstone et al., 2013; Stampoultzis et al., 2021). As the fundamental component for engineered cartilage construction, seed cells are required to possess abundant sources, strong proliferative ability, stable chondrogenic differentiation potential and low immunogenicity (Liu et al., 2021a). Rat adipose-derived stem cells (ADSCs), a type of multipotent mesenchymal stem cells (MSCs), can be easily isolated from adipose tissues with minimal trauma and abundant reserves. Under specific induction conditions, ADSCs can directionally differentiate into chondrocytes and secrete cartilaginous extracellular matrix. Therefore, ADSCs are promising seed cells for cartilage tissue engineering and have been widely adopted in experimental studies on cartilage regeneration (Liu et al., 2021a; Zuk et al., 2001; Li et al., 2021; Zhao et al., 2025).

As carriers for seed cells, scaffolds are supposed to provide a stable three-dimensional (3D) structure for cell adhesion, proliferation and differentiation, along with favorable biocompatibility, biodegradability and appropriate mechanical properties. Sodium alginate (NaAlg), a natural polysaccharide hydrogel, features wide availability, excellent biocompatibility and tunable degradation rate. It can form 3D scaffolds via calcium ion crosslinking, which encapsulate seed cells gently and maintain the spherical phenotype of chondrocytes, thereby creating an *in vivo*-like physiological microenvironment for cell growth. Moreover, the mechanical properties of NaAlg hydrogel scaffolds can be optimized by adjusting material concentrations, making it one of the most commonly used scaffolds in cartilage tissue engineering (Jarrah et al., 2023; Xie et al., 2022; Chen et al., 2024; Liu et al., 2022a). Accumulated studies have demonstrated that NaAlg hydrogel scaffolds effectively support the adhesion and proliferation of mesenchymal stem cells and facilitate the secretion of cartilaginous matrix (Zahedi Tehrani et al., 2024; Chen et al., 2025). However, pure NaAlg hydrogel scaffolds still have deficiencies in cell adhesion and mechanical strength, and thus need to be combined with seed cells and inductive factors to improve chondrogenic outcomes.

Inductive factors are key regulators that drive the directional chondrogenic differentiation of seed cells and promote the synthesis of cartilaginous matrix. Cartilage-derived morphogenetic protein-1 (CDMP-1), also known as growth differentiation factor 5 (GDF-5) or bone morphogenetic protein 14 (BMP-14), belongs to the bone morphogenetic protein (BMP) family and the transforming growth factor-β (TGF-β) superfamily. It plays a vital regulatory role in the development and regeneration of cartilage and bone (Chang et al., 1994). Existing evidence has verified that CDMP-1 can specifically recruit mesenchymal stem cells towards chondrogenic progenitor cells and accelerate their differentiation into chondrocytes (Wang et al., 2018; Zhang et al., 2011; Feng et al., 2008). It upregulates the expression of chondrogenic markers including SOX9, collagen II (COL-II) and aggrecan (ACAN), maintains the chondrocyte phenotype and inhibits chondrocyte hypertrophy. In addition, CDMP-1 alleviates inflammatory responses and creates a favorable microenvironment for cartilage regeneration, exhibiting great application potential in the repair of cartilage injuries.

To date, systematic investigations on engineered cartilage constructed by CDMP-1 combined with ADSCs/NaAlg composite scaffolds remain insufficient. On this basis, the present study adopted tissue engineering techniques to explore the feasibility and efficacy of this composite scaffold for engineered cartilage construction, aiming to provide experimental evidence and new strategies for the clinical repair of cartilage defects via tissue engineering.

## 2 Materials and methods

### 2.1 Ethical statement

All experimental protocols were approved by the Institutional Animal Care and Use Committee of Fujian Medical University (No. IACUC-FJMU-20230739) and conducted in accordance with the Guidelines for the Care and Use of Laboratory Animals.

### 2.2 *In vitro* cell experiments

#### 2.2.1 Cell isolation and culture

Eight-week-old healthy male specific-pathogen-free (SPF) Sprague–Dawley (SD) rats (n = 20) were anesthetized with 0.2% pentobarbital sodium (50 mg/kg) by intraperitoneal injection. Bilateral inguinal adipose tissues were excised and minced thoroughly. The tissues were digested with 0.1% collagenase type I (Sigma, C0130), and the digestion was terminated using DMEM/F-12 basal medium. The cell suspension was sequentially filtered through 100 μm nylon cell strainers (Nest, 258365) to remove tissue debris and centrifuged at 1,200 rpm for 5 min. After discarding the supernatant, the cell pellet was resuspended with DMEM/F-12 medium containing 10% FBS (Gibco, 10099-141). The cell density was adjusted to 1 × 10^6^ to 1 × 10^7^ cells/mL, and cells were seeded in T25 culture flasks. Cells were incubated in a humidified incubator (Thermo Fisher, 371) at 37 °C with 5% CO_2_. The medium was first replaced 24 h post-seeding to remove non-adherent cells and refreshed every 2–3 days thereafter. Cell growth was observed under an inverted microscope (BD, United States). When cells reached 80%–90% confluence, the first passage was conducted. Cells were digested with 0.25% trypsin-EDTA (Gibco, 25200-056) and subcultured at a ratio of 1:2 to 1:3. Passage 3 ADSCs were used for all subsequent experiments to ensure cell source homogeneity and experimental result stability.

#### 2.2.2 ADSC characterization

Passage 3 ADSCs were harvested and digested with 0.25% trypsin-EDTA to prepare single-cell suspensions. The cell density was adjusted to 1 × 10^6^ cells/mL. A 500-μL cell suspension was incubated with 5 μL each of FITC-CD29, PE-CD31, PE-CD44, PE-CD45, and PE-CD105 monoclonal antibodies (Immunoway, United States). FITC-IgG and PE-IgG were used as isotype controls. After incubation in the dark at room temperature for 30 min, the cells were washed with PBS and centrifuged at 1,200 rpm for 5 min, and the supernatant was removed. The cells were resuspended in 500 μL PBS and analyzed using flow cytometry (Beckman Coulter, United States), with FlowJo software used to analyze CD29, CD31, CD44, CD45, and CD105 expressions.

#### 2.2.3 Material preparation and grouping

**FIGURE 1:**
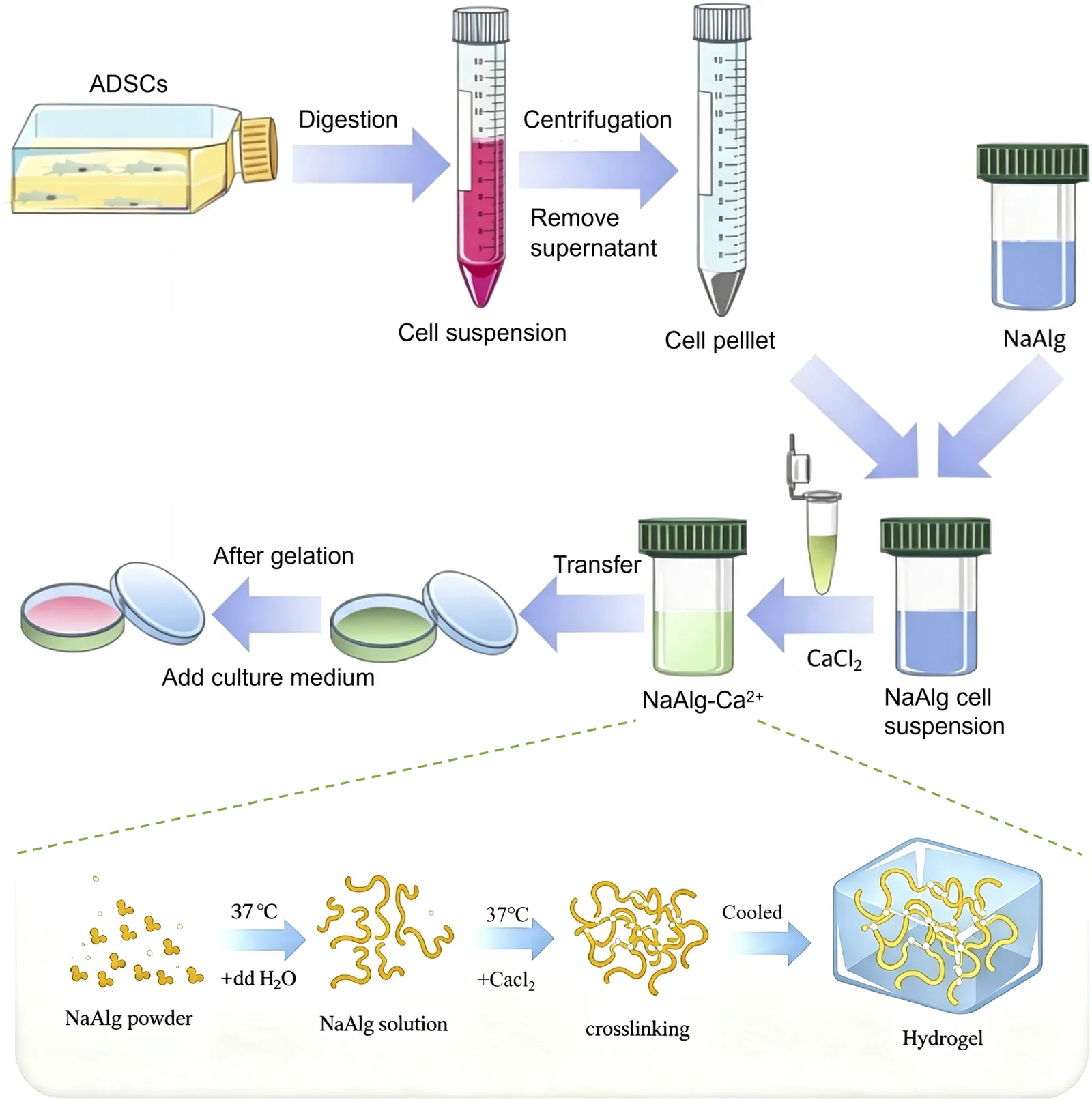
Schematic of NaAlg hydrogel fabrication and 3D culture of encapsulated ADSCs.

Passage 3 ADSCs were adjusted to a density of 1 × 10^8^ cells/L and mixed thoroughly with 2 mL of 0.5% sodium alginate (Sigma, United States) solution containing CDMP-1(PeproTech, 120-01). The mixture was dropped into 24-well plates preloaded with 1 mL of 2% CaCl_2_ solution at a volume of 50 μL per droplet (approximately 2500 cells per droplet), with a total volume of 200 μL per well and 12 replicate wells. The mixture was allowed to form hydrogel at room temperature (Figure 1). Sodium alginate hydrogel combined with ADSCs without CDMP-1 was prepared in the same manner. For chondrogenic induction, the CDMP-1 + ADSCs + NaAlg group was treated with 1.5 μg/L CDMP-1. The ADSCs + NaAlg group was cultured in chondrogenic induction medium consisting of DMEM/F-12 containing 10% FBS, 10^−7^ mol/L dexamethasone, 6.25 mg/L insulin, 50 mg/L ascorbic acid and 40 mg/L vitamin C (Sigma, United States). Meanwhile, passage 3 ADSCs were seeded in 6-well plates with pre-placed coverslips at a density of 1 × 10^6^ cells/mL, which were divided into the ADSCs group and CDMP-1 + ADSCs group.

#### 2.2.4 Biocompatibility evaluation

Live/dead cell fluorescent staining, cell morphology fluorescent staining (phalloidin cytoskeleton staining), Transwell migration assay, and scratch assay were performed to evaluate the biocompatibility of the scaffolds. For indirect co-culture, ADSCs were seeded in the lower chamber of Transwell inserts with 0.4 μm pores, and scaffolds were placed in the upper chamber.

1. Live/dead cell fluorescent staining: A Calcein AM/PI Fluorescent Staining Kit (Elabscience, Wuhan, China) was used to observe ADSC growth activity after different treatments. Calcein AM detects intracellular esterase activity, whereas PI detects cell membrane integrity for biocompatibility assessment. Cells were seeded at 1 × 10^4^ cells/well in 24-well plates. After cell attachment, grouping was performed as described in section 2.2.3. After 3 and 7 days of culture, cells were carefully washed three times with PBS, fixed with 4% paraformaldehyde (PFA) for 15 min at room temperature, and washed again with PBS (three times, 3 min each). Following the Calcein AM/PI Live/Dead Cell Double Staining Kit instructions, 250 μL 1% Calcein AM/PI working solution was added to each well of 24-well plates for live/dead cell fluorescent staining for 30 min at room temperature in the dark, followed by PBS washing. Images were captured using an inverted fluorescence microscope (Carl Zeiss, Germany), with live cells showing green fluorescence (Calcein AM-positive) and dead cell nuclei showing red fluorescence (PI-positive). Each experiment included four technical replicates per treatment group, and all experiments were independently repeated four times (n = 4 independent experiments).
2. Cell morphology fluorescent staining: Phalloidin/DAPI cytoskeleton staining was used to study ADSC morphology under different treatments. Phalloidin specifically binds cellular actin to label cell contours, whereas DAPI binds double-stranded DNA in cell nuclei. Staining working solution was prepared by adding 5 μL rhodamine phalloidin (Immunoway, United States) into 1 mL 5× dilution buffer. Cells were seeded at 1 × 10^4^ cells/well in 24-well plates. After cell attachment, grouping was performed as described in section 2.2.3. After 3 and 7 days of culture, cells were carefully washed three times with PBS, fixed with 4% PFA for 15 min at room temperature, and washed again with PBS, permeabilized with 0.1% Triton X-100 (Sigma, United States) for 10 min at room temperature, and washed again with PBS, and blocked with 1% BSA (Sigma, United States) for 30 min at room temperature. FITC-conjugated phalloidinworking solution (5 μg/mL) was added, followed by incubation at room temperature for 30 min in the dark to stain F-actin. After PBS washing (three times, 3 min each), DAPI (100 ng/mL) mounting medium was used for nuclear counterstaining and mounting. Cell morphology, spreading, and F-actin distribution were observed using an inverted fluorescence microscope (Carl Zeiss, Germany) with image recording. Each experiment included four technical replicates per treatment group, and all experiments were independently repeated four times (n = 4 independent experiments).
3. Transwell migration assay: Transwell chambers with 8.0 μm pore-sized polycarbonate membrane inserts (Corning, United States) were used to evaluate the effects of different treatments on ADSC migration ability. ADSCs in the logarithmic growth phase were digested and resuspended in serum-free MDMEM/F-12 medium at 1 × 10^5^ cells/mL density. A 200-μL cell suspension (1 × 10^5^ cells) was seeded in Transwell upper chambers. Lower chambers contained 500 μL medium with different treatments (grouping described in section 2.2.3). Culture plates were placed in 37 °C, 5% CO_2_ incubators for 24 h and 48 h. After culture, upper chambers were removed, and non-migrated cells on the inner surfaces of the upper chamber were gently wiped with moist cotton swabs. Cells that migrated to the membrane outer sides of the lower chamber were fixed with 4% PFA for 30 min at room temperature and then stained with 1% crystal violet solution (Solarbio, Beijing, China) for 15 min. After washing excess dye with PBS (three times, 3 min each), images were captured under an inverted microscope, and migrated cells were counted. Each experiment included four technical replicates per treatment group, and all experiments were independently repeated four times (n = 4 independent experiments).
4. Scratch assay: Scratch assays evaluated effects of different treatments on ADSC lateral migration ability. ADSCs in the logarithmic growth phase were collected and seeded at 1 × 10^5^ cells/well in 24-well plates and then cultured until approximately 90%–100% confluence to form monolayers. Sterile 200-μL pipette tips were used to rapidly scratch across cell monolayer centers, creating uniform-width scratches. Cells were gently washed 2–3 times with PBS to remove detached cells and debris. Medium containing different treatments (grouping described in section 2.2.3) was then added to each well. Scratch area images were captured at the same positions at 0 h (immediate), 24 h, and 48 h using an inverted microscope. ImageJ software (National Institutes of Health, United States) was used to measure and calculate the scratch area, with cell migration ability assessed by comparing scratch closure degrees at different time points. Each experiment included four technical replicates per treatment group, and all experiments were independently repeated four times (n = 4 independent experiments).

#### 2.2.5 Cell proliferation assay

Cells were harvested at days 1, 3, 5, 7 and 9 after chondrogenic induction for each group (grouping described in section 2.2.3). The hydrogel carriers were dissolved using 55 mmol/L sodium citrate, and cells were collected by centrifugation and transferred into 96-well plates. After 24 h of cell attachment, the medium was removed, and 100 μL of complete medium containing 10% CCK-8 (Beyotime, Shanghai, China) was added to each well, followed by 1–2 h of incubation. Absorbance (OD value) at 450 nm was measured using a multifunctional microplate reader (BioTek, United States). A growth curve was plotted using the mean OD values of six wells as the ordinate and culture time as the abscissa. Each experiment included six technical replicates per treatment group, and all experiments were independently repeated four times (n = 6 independent experiments).

#### 2.2.6 Immunohistochemical staining

Cells were harvested at days 1, 3, 5, 7 and 9 after chondrogenic induction for each group (grouping described in section 2.2.3). Hydrogels were dissolved with 55 mmol/L sodium citrate, and cells were collected by centrifugation and seeded in 24-well plates. After 24 h of cell attachment, the medium was removed, cells were fixed with 4% PFA for 20 min and washed with PBS. Cells were permeabilized with 0.1% Triton X-100 at room temperature for 15 min and washed again with PBST. After blocking with 5% goat serum (Meilun, Dalian, China) for 30 min at room temperature, cells were incubated with mouse monoclonal primary antibody against COL-II overnight at 4 °C. Following PBST washing, cells were incubated with HRP-conjugated goat anti-mouse secondary antibody at 37 °C for 1 h in the dark. After PBST rinsing, DAB chromogenic solution (Immunoway, United States) was added for color development for 10 min at room temperature, and the reaction was terminated with running water. Staining results were observed and photographed under an inverted microscope. Each experiment included four technical replicates per treatment group, and all experiments were independently repeated four times (n = 4 independent experiments). For quantitative analysis of immunohistochemical staining, three random high-power fields (×100 magnification) per well were photographed using standardized exposure settings. Positive staining intensity was quantified using ImageJ software, with the following protocol: images were converted to RGB color images, and brown DAB-positive staining was isolated using color threshold analysis. The integrated optical density (IOD) of positive staining was measured and normalized to the total area of each field. The mean IOD/area ratio was calculated for each group, with three sections per well and four wells per group analyzed for statistical evaluation.

#### 2.2.7 Toluidine blue staining

Cells were harvested at days 1, 3, 5, 7 and 9 after chondrogenic induction for each group (grouping described in section 2.2.3). Hydrogels were dissolved with 55 mmol/L sodium citrate, and cells were collected by centrifugation and seeded in 24-well plates. After 24 h of cell attachment, the medium was removed, cells were fixed with 4% PFA for 15 min and rinsed with PBS. A total of 400 μL toluidine blue staining solution was added to each well, and cells were incubated at room temperature for 15 min. Staining outcomes were observed and photographed under an inverted microscope. Each experiment included four technical replicates per treatment group, and all experiments were independently repeated four times (n = 4 independent experiments). For quantitative analysis of toluidine blue staining, three random high-power fields (×100 magnification) per well were photographed using standardized exposure settings. Positive staining intensity was quantified using ImageJ software, with the following protocol: images were converted to RGB color images, and brown DAB-positive staining was isolated using color threshold analysis. The integrated optical density (IOD) of positive staining was measured and normalized to the total area of each field. The mean IOD/area ratio was calculated for each group, with three sections per well and four wells per group analyzed for statistical evaluation.

#### 2.2.8 Immunofluorescence staining

Cells were harvested at days 7 and 14 after chondrogenic induction for each group (grouping described in section 2.2.3). Hydrogels were dissolved with 55 mmol/L sodium citrate, and cells were collected by centrifugation and seeded in 6-well plates. After 24 h of cell attachment, the medium was removed, and cells were washed with PBS and fixed with 4% PFA for 15 min. Cells were permeabilized with 0.1% Triton X-100 for 15 min at room temperature and washed with PBST. After blocking with 5% goat serum for 30 min, cells were incubated with mouse monoclonal primary antibodies against SOX9, COL-II and ACAN (Immunoway, United States) overnight at 4 °C. After PBST washing, cells were incubated with FITC-conjugated goat anti-mouse secondary antibody at 37 °C for 1 h in the dark. Cells were washed with PBST and mounted with fluorescence antifade mounting medium containing DAPI. Slides were dried in the dark and stored at 4 °C before observation and imaging using an inverted fluorescence microscope (Leica, Germany). Immunofluorescence images were captured under an inverted fluorescence microscope with identical acquisition parameters among all groups. The mean fluorescence intensity (MFI) was quantified using ImageJ software. Briefly, single cells were manually outlined, and background fluorescence was subtracted. Relative fluorescence intensity was calculated by normalizing the corrected MFI of each group to the control group. Each experiment included four technical replicates per treatment group, and all experiments were independently repeated four times (n = 4 independent experiments).

#### 2.2.9 Quantitative real-time PCR (qRT-PCR)

qRT-PCR was used to analyze mRNA expression levels of chondrogenesis-related genes. Cells were harvested at days 7 and 14 after chondrogenic induction for each group (grouping described in section 2.2.3). Hydrogels were dissolved with 55 mmol/L sodium citrate, and cells were collected by centrifugation and seeded in 6-well plates. After cell attachment, the medium was removed, and the total RNA was extracted using TRIzol reagent (Thermo Fisher, United States). RNA concentration and purity were measured using a NanoDrop spectrophotometer (A260/A280 ratio between 1.8 and 2.0). One microgram of total RNA was reverse-transcribed into cDNA using PrimeScript™ RT Master Mix. Diluted cDNA was used as a template for amplification using SYBR® Premix Ex Taq™ II (TaKaRa, Japan) on a real-time fluorescent quantitative PCR instrument (Thermo Fisher, United States). Target genes included key chondrogenesis differentiation transcription factors *SOX9* (SRY-box transcription factor 2), *COL2A1* (Collagen type II alpha 1), and *ACAN* (Aggrecan). *GAPDH* (glyceraldehyde-3-phosphate dehydrogenase) served as an internal reference gene for normalization. Primer sequences were designed using Primer Premier 5.0 software and are listed in Table 1. All primers were synthesized by Sangon Biotech (Shanghai, China) and validated for specificity and efficiency. Each sample had three technical replicates. The relative expression of target genes was calculated using the 2^−ΔΔCT^ method.

**TABLE 1:** Primer sequences used for quantitative real-time PCR analysis.

Gene	Forward primer (5′-3′)	Reverse primer (5′-3′)	Product size (bp)
*SOX9*	TAA​ATT​CCC​AGT​GTG​CAT​CC	GCA​CCA​GGG​TCC​AGT​CAT​A	182 bp
*COL2A1*	CCT​GGA​CCC​CGT​GGC​AGA​GA	CAG​CCA​TCT​GGG​CTG​CAA​AG	156 bp
*ACAN*	AGG​ATG​GCT​TCC​ACC​AGT​GC	TGC​GTA​AAA​GAC​CTC​ACC​CTC​C	179 bp
*GAPDH*	GCAAGTTCAACGGCACAG	CGC​CAG​TAG​ACT​CCA​CGA​C	142 bp

### 2.3 Statistical analysis

All data were analyzed using SPSS 26.0 software (IBM, Armonk, NY, United States). Data were presented as mean ± standard deviation. One-way ANOVA followed by Tukey’s multiple comparison test was adopted for statistical analysis. A value of *P* < 0.05 was considered statistically significant. The statistical methods in this study have been reviewed by biostatisticians from Ningde Normal University.

## 3 Results

### 3.1 ADSC culture and characterization

**FIGURE 2:**
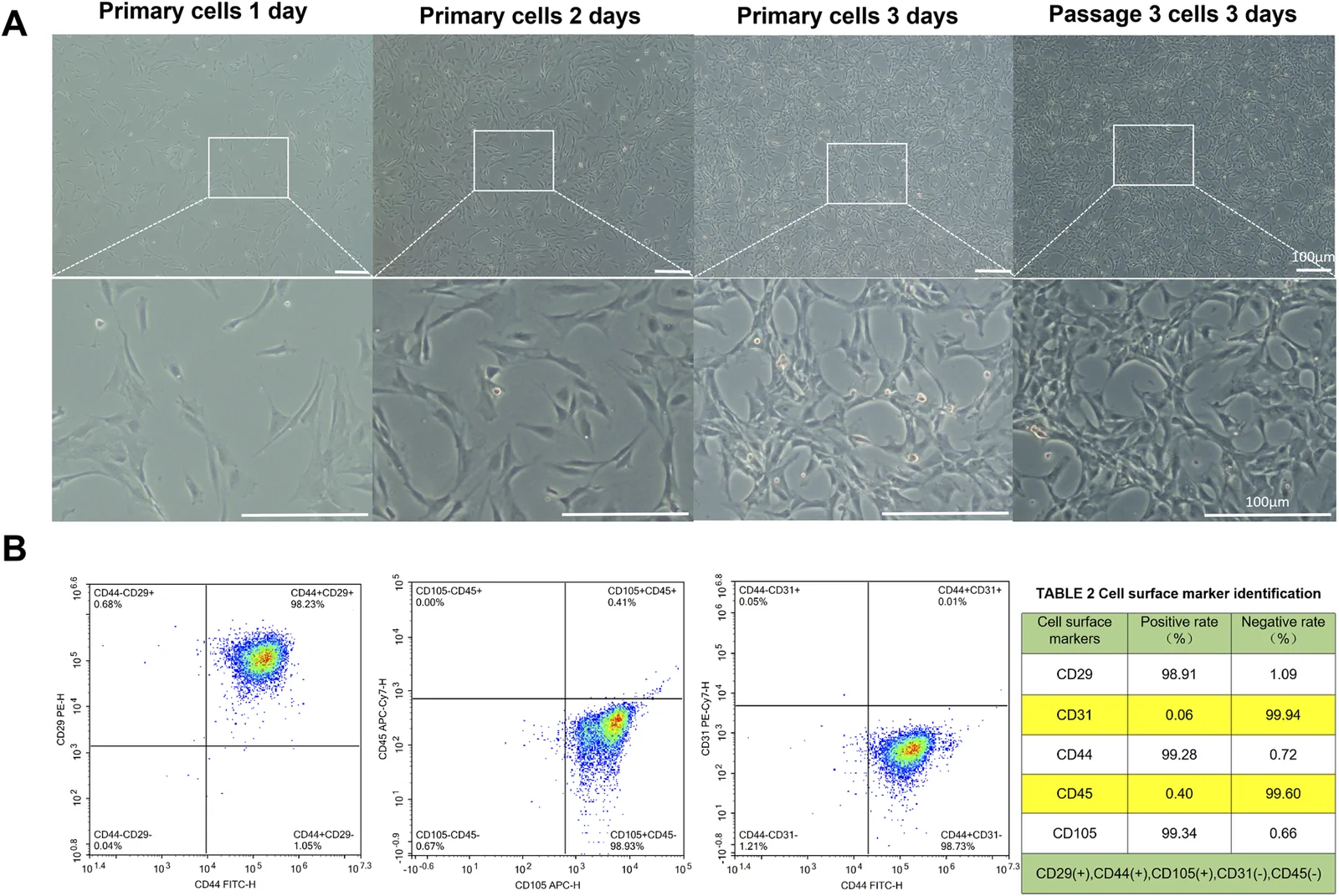
ADSCs Morphology and Phenotypic Characteristics: **(A)** Primary cells at first medium change with unattached cells eliminated; most cells were short spindle-shaped and polygonal. Primary cells at day 2 of culture, 60% confluent with elongated spindle morphology. Primary cells at day 3 of culture, 90% confluent, showing typical elongated spindle shape and vortex-like arrangement. Passage 3 cells cultured for 3 days with consistent elongated spindle shape and vortex-like distribution. **(B)** Identification of ADSCs surface markers by flow cytometry, the results showed that CD29, CD44 and CD105 were positively expressed, with positive rates of 98.91%, 99.28% and 99.34%, respectively. CD31 and CD45 were negatively expressed, with negative rates of 99.94% and 99.60%. Scale bar = 100 µm.

The freshly isolated cells presented a small round morphology and began to adhere to the culture surface at 24 h after incubation. The non-adherent cells remained round or spherical, which were removed during the first medium replacement. Most adherent cells exhibited a short spindle or polygonal shape (Figure 2A). With prolonged culture, the cell number increased gradually, and the cells displayed a typical long spindle morphology with colony and cluster growth patterns (Figure 2A). At 3–4 days of culture, the cells reached 80%–90% confluence and arranged in a swirling pattern (Figure 2A). After subculture, the proliferation rate of ADSCs was accelerated. Cell passaging could be performed every 2–3 days, and the cells maintained stable growth during long-term culture (Figure 2A).

Flow cytometry analysis was conducted to characterize surface biomarkers (CD29, CD31, CD44, CD45, CD105) of passage 3 ADSCs. The results showed that CD29, CD44 and CD105 exhibited high positivity at 98.91%, 99.28% and 99.34%, whereas CD31 and CD45 displayed extremely low positive rates of 0.06% and 0.4% (Figure 2B). These results demonstrated that the harvested passage 3 ADSCs possessed typical MSC immunophenotypes, with abundant expression of mesenchymal markers and negligible expression of endothelial marker CD31 and hematopoietic marker CD45. The surface antigen profile was consistent with the phenotypic characteristics of ADSCs, confirming that the cells isolated via type I collagenase digestion, centrifugation and adherent culture were ADSCs after subculture. The cell phenotype meets the requirements for follow-up experiments.

### 3.2 Biocompatibility of NaAlg hydrogel scaffold

Excellent biocompatibility is a fundamental prerequisite for the *in vivo* application of biomaterials. In this study, live/dead cell staining, cytoskeleton staining, Transwell migration assay, and scratch assay were performed to evaluate the effects of composite scaffolds on ADSCs.

**FIGURE 3:**
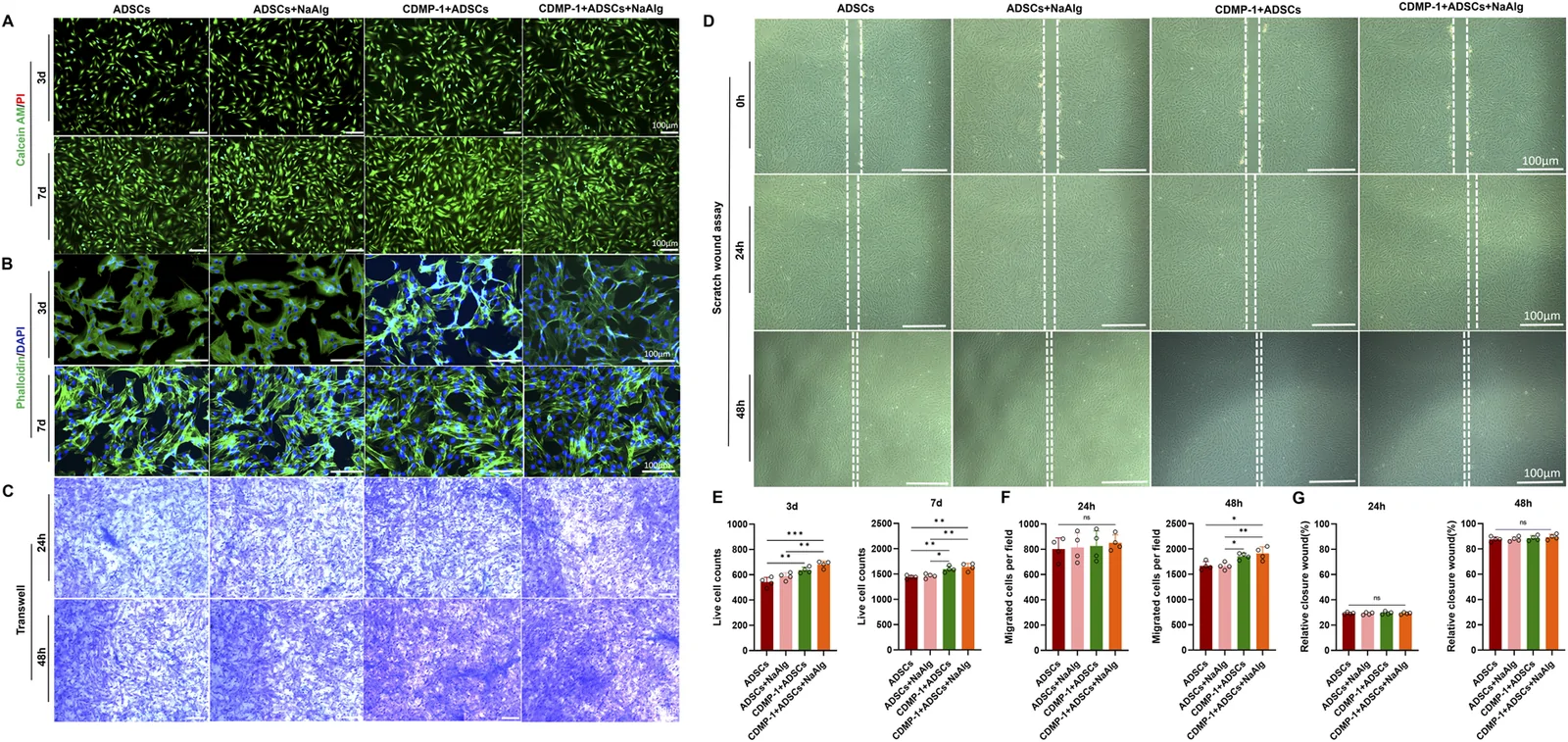
Biological safety evaluation of sodium alginate hydrogel was performed and CDMP-1 promotes ADSCs proliferation, improves cellular morphology, and elevates migration capacity: **(A,E)** Calcein AM/PI live/dead staining at days 3 and 7, demonstrating enhanced cell viability with CDMP-1 treatment. Green: live cells; red: dead cells. Quantitative analysis of the live cells. **(B)** Phalloidin/DAPI staining revealing improved cell spreading and F-actin organization. Green: F-actin; blue: nuclei. **(C,F)** Transwell migration assay demonstrating enhanced chemotactic migration. Representative images of migrated cells stained with crystal violet and quantitative cell counts. **(D,G)**scratch wound healing assay showing accelerated gap closure with CDMP-1 treatment at 0, 24, and 48 h. Quantitative analysis of the relative closure wound area percentage. Scale bar = 100 µm. The significance of biologically independent samples (n = 4) was calculated by ANOVA followed by Tukey’s multiple comparisons. Data were presented as mean ± SD. Significant differences between groups were indicated as *P < 0.05, **P < 0.01, ***P < 0.001, and ns: no significance.

Live/dead staining (Figures 3A,E) showed that, after 3 and 7 days of co-culture, no significant differences in cell viability were observed when comparing ADSCs vs. ADSCs + NaAlg and ADSCs + CDMP-1 vs. ADSCs + CDMP-1 + NaAlg under fixed CDMP-1 treatment conditions (*P* > 0.05). In contrast, with NaAlg treatment conditions fixed, comparisons of ADSCs vs. ADSCs + CDMP-1 and ADSCs + NaAlg vs. ADSCs + CDMP-1 + NaAlg exhibited statistically significant differences (*P* < 0.01). These results demonstrated that CDMP-1 effectively promoted the proliferation and chondrogenic differentiation of ADSCs, while the NaAlg hydrogel scaffold exerted a limited effect on cell proliferation.

Cytoskeleton staining (Figure 3B) further revealed that cells in all groups retained normal morphology and spreading capacity after 7 days of co-culture, confirming that NaAlg and CDMP-1 could maintain cell viability.

To evaluate the regulatory effects of NaAlg and CDMP-1 on the migratory capacity of ADSCs, two complementary assays, the Transwell migration assay and scratch assay, were employed in this study. Transwell migration assays (Figures 3C,F) showed that no intergroup differences in the number of migrated cells were detected at 24 h (*P* > 0.05). At 48 h, however, the CDMP-1 + ADSCs group and CDMP-1 + ADSCs + NaAlg group (CDMP-1-treated groups) exhibited markedly more migrated cells than the ADSCs group and ADSCs + NaAlg group (non-CDMP-1-treated groups), with statistically significant differences (*P* < 0.05). Similarly, no significant differences in migrated cell counts were found between the NaAlg-treated groups (ADSCs + NaAlg and CDMP-1 + ADSCs + NaAlg) and non-NaAlg-treated groups (ADSCs and CDMP-1 + ADSCs) (*P* > 0.05). These results suggested that CDMP-1 exerted a chemotactic effect on ADSCs, while the scaffold material possessed no chemotactic activity. Nevertheless, scratch assays (Figures 3D,G) demonstrated that the wound closure rate showed no statistically significant differences among all treatment groups at 24 h and 48 h after scratching (*P* > 0.05). This implied that the migratory effect of CDMP-1 on ADSCs may exist within a specific concentration window. The results from the Transwell migration assay and scratch assay cannot fully confirm that CDMP-1 elicits prominent chemotactic and pro-migratory effects on ADSCs. Further investigations with serially diluted CDMP-1 concentrations are required to verify this conclusion.

Collectively, these results verify that the composite scaffolds possess favorable biocompatibility and exert no cytotoxicity against ADSCs, thereby serving as an ideal cell carrier.

### 3.3 Detection of proliferative activity of ADSC after chondrogenic induction via CCK-8 assay

**FIGURE 4:**
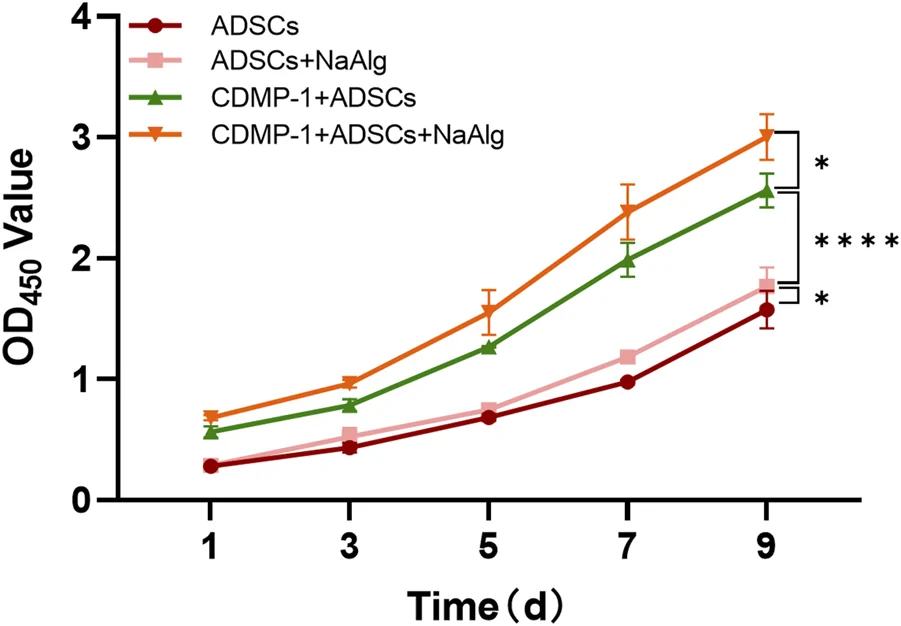
Proliferative activity of ADSCs following chondrogenic induction. Cell proliferative activity in each group was detected via CCK-8 assay. Optical density (OD) values at 450 nm were measured at different time points to plot cell growth curves. Significant differences in cell proliferative activity were observed among all groups (*P < 0.05, ****P < 0.0001).

The proliferative activity of ADSCs in each group was determined using the CCK-8 assay at days 1, 3, 5, 7 and 9 after chondrogenic induction. A growth curve was plotted with culture time as the abscissa and the mean optical density (OD) values of six replicates as the ordinate (Figure 4). All groups exhibited favorable cell proliferation. The logarithmic growth phase occurred from day 3 to day 7, and cell proliferation entered a plateau phase at day 9. Statistically significant differences were observed among groups at days 3, 5, 7 and 9 (*P* < 0.0001, Figure 4). ADSCs cultured with NaAlg hydrogel scaffolds or CDMP-1 showed accelerated growth compared with the pure ADSCs group (*P* < 0.05 vs. *P* < 0.0001). CDMP-1 exerted a more prominent promotive effect on ADSCs proliferation than NaAlg (*P* < 0.0001). Notably, the CDMP-1 + ADSCs + NaAlg group presented the highest proliferation rate (*P* < 0.001). These findings demonstrated that both CDMP-1 and NaAlg could promote the proliferation of ADSCs, and their combination exerted a synergistic enhancing effect.

### 3.4 Detection of chondrogenic differentiation markers

**FIGURE 5:**
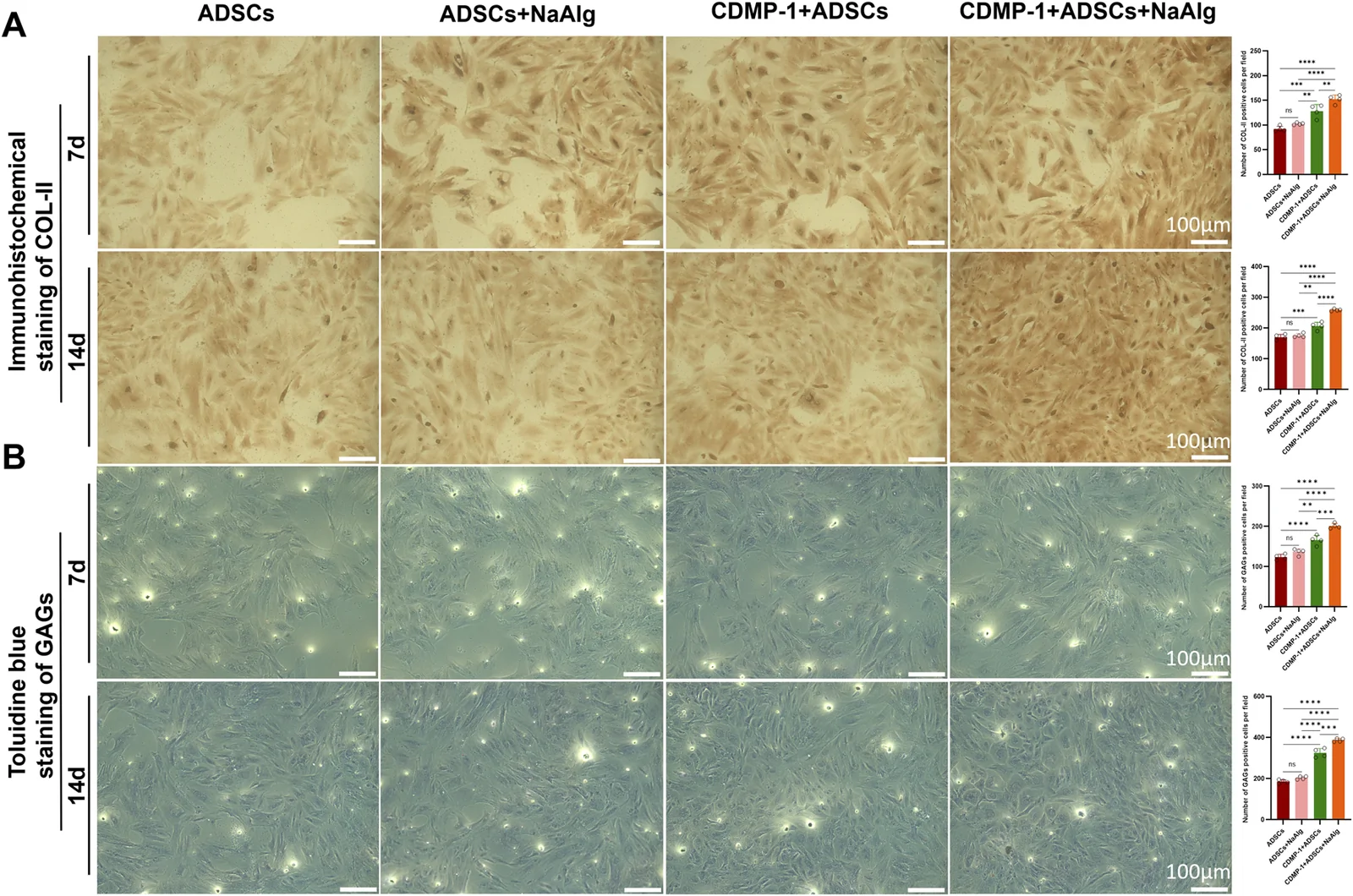
*In vitro* chondrogenic differentiation ability of ADSCs: **(A)** Immunohistochemical staining and quantitative analysis of COL-II in each group after 7 and 14 days of induction. **(B)** Toluidine blue staining and quantitative analysis of GAGs in each group after 7 and 14 days of induction. Scale bar = 100 µm. The significance of biologically independent samples (n = 4) was calculated by ANOVA followed by Tukey’s multiple comparisons. Data were presented as mean ± SD. Significant differences between groups were indicated as **P < 0.01, ***P < 0.001, ****P < 0.0001, and ns: no significance.

**FIGURE 6:**
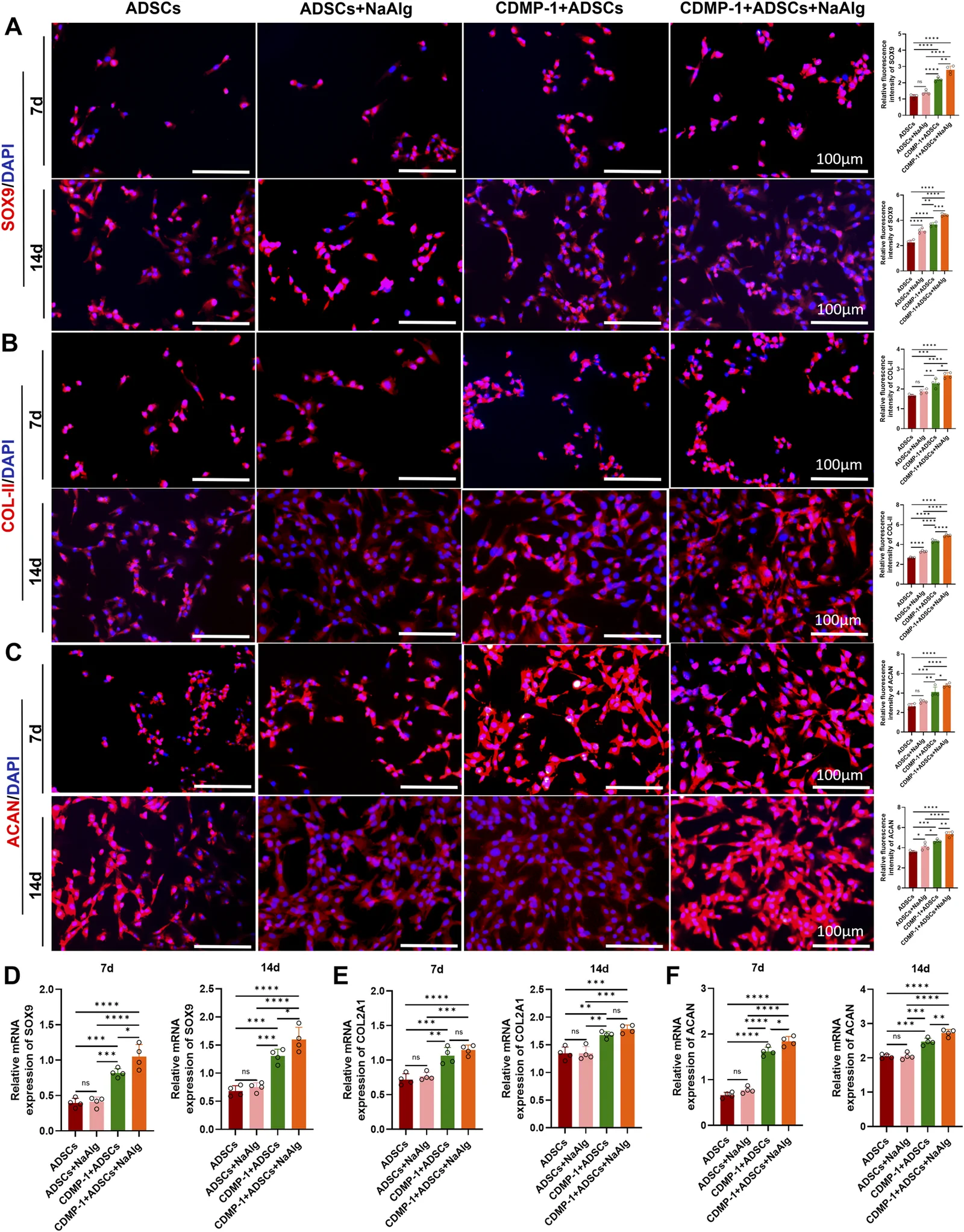
Immunofluorescence staining and qRT-PCR were used to detect the protein and gene expression levels of SOX-9,COL-II and ACAN: **(A)** Immunofluorescence and quantitative analysis of SOX-9 expression in different groups after 7 and 14 days of chondrogenic induction. **(B)** Immunofluorescence and quantitative analysis of COL-II expression in different groups after 7 and 14 days of chondrogenic induction. **(C)** Immunofluorescence and quantitative analysis of ACAN expression in different groups after 7 and 14 days of chondrogenic induction. **(D)** Relative mRNA expression of *SOX-9* gene in different groups at days 7 and 14 after chondrogenic induction. **(E)** Relative mRNA expression of *COL2A1* gene in different groups at days 7 and 14 after chondrogenic induction. **(F)** Relative mRNA expression of *ACAN* gene in different groups at days 7 and 14 after chondrogenic induction. Scale bar = 100 µm. The significance of biologically independent samples (n = 4) was calculated by ANOVA followed by Tukey’s multiple comparisons. Data were presented as mean ± SD. Significant differences between groups were indicated as *P < 0.05, **P < 0.01, ***P < 0.001, ****P < 0.0001, and ns: no significance.

Immunohistochemical staining for COL-II and toluidine blue staining for sulfated glycosaminoglycans (GAGs) were both positive in all groups at days 7 and 14 after chondrogenic induction. Under an inverted phase-contrast microscope, COL-II was stained brownish-yellow in the cytoplasm, with yellow staining observed in the extracellular regions. GAGs were stained blue in the cytoplasm and surrounding areas (Figure 5). The expression levels of COL-II and GAGs were relatively low on day 7, and markedly increased on day 14 in all groups. The expression of these two markers was significantly higher in the CDMP-1 + ADSCs group and CDMP-1 + ADSCs + NaAlg group (CDMP-1-treated groups) than in the ADSCs group and ADSCs + NaAlg group (control groups) (*P* < 0.001), indicating that CDMP-1 could promote chondrogenic differentiation of ADSCs. Among the four groups, the CDMP-1 + ADSCs + NaAlg group showed the strongest expression of COL-II and GAGs, with statistically significant differences (*P* < 0.01). This result suggested that CDMP-1 combined with sodium alginate hydrogel exerted a synergistic effect on facilitating chondrogenic differentiation of ADSCs. See Figure 6 for details.

### 3.5 Detection of chondrogenesis-related proteins expression by immunofluorescence staining

As shown in Figures 6A–C, the protein levels of SOX9, COL-II and ACAN increased gradually in all groups at days 7 and 14 post-induction. The expression of SOX9 protein in the CDMP-1 + ADSCs + NaAlg group was significantly higher than that in the other three groups at both time points (*P* < 0.01). Similarly, COL-II and ACAN protein expression in this group was also markedly elevated compared with the other three groups (*P* < 0.05). At day 7, no significant differences in SOX9, COL-II and ACAN protein expression were found between the ADSCs group and the ADSCs + NaAlg group (*P* > 0.05), whereas significant differences were detected at day 14 (*P* < 0.05).

### 3.6 Detection of chondrogenesis-related genes expression by qRT-PCR

As presented in Figures 6D–F, the mRNA expression of *SOX9, COL2A1* and *ACAN* was upregulated over time in all groups at days 7 and 14 after chondrogenic induction. The CDMP-1 + ADSCs + NaAlg group exhibited higher *SOX9* mRNA levels than the other three groups at both time points (*P* < 0.05), the same as *ACAN*. For *COL2A1* mRNA, its expression in the CDMP-1 + ADSCs + NaAlg group was higher than that in the remaining two groups (*P* < 0.001), while no significant difference was observed when compared with the CDMP-1 + ADSCs group (*P* > 0.05). In addition, there were no statistically significant differences in the mRNA expression of *SOX9*, *COL2A1* and *ACAN* between the ADSCs group and the ADSCs + NaAlg group at either day 7 or day 14 (*P* > 0.05).

## 4 Discussion

Articular cartilage is devoid of blood vessels, nerves and lymphatic vessels, resulting in extremely limited self-repair capacity. Physiological regeneration is hardly achieved after trauma or degeneration, which remains a major challenge in clinical orthopedics and sports medicine. Conventional clinical repair strategies are compromised by multiple drawbacks, including fibrocartilage formation, donor tissue shortage and mismatched biomechanical properties, failing to accomplish functional cartilage repair. Tissue engineering technology integrates seed cells, 3D scaffolds and inductive factors to precisely mimic the *in vivo* microenvironment of cartilage growth, offering a novel strategy for the physiological repair of cartilage defects. In this study, rat ADSCs were used as seed cells, NaAlg hydrogel scaffold as the 3D scaffold, and cartilage-derived morphogenetic protein-1 (CDMP-1) as the chondrogenic inducer. We systematically investigated the biocompatibility and *in vitro* chondrogenic differentiation efficiency of this ternary composite system. This study aims to optimize the cartilage tissue engineering strategy and provide experimental evidence for subsequent *in vivo* repair experiments and clinical translation. Most previously published studies on CDMP-1 or alginate-based scaffolds adopted a single-factor design lacking systematic assessment, or centered on bone marrow mesenchymal stem cells (BMSCs). No prior reports have described the triple combination of ADSCs, CDMP-1 and NaAlg hydrogel scaffolds. Herein, we innovatively constructed an *in vitro* chondrogenic differentiation system by combining CDMP-1, NaAlg and ADSCs, followed by systematic characterization, thereby promoting the development of current tissue engineering strategies.

The selection and viability maintenance of seed cells are the foundation for constructing tissue-engineered cartilage. As a type of MSCs, ADSCs possess the advantages of abundant sources, minimally invasive acquisition, strong proliferative capacity and low immunogenicity, along with stable chondrogenic differentiation potential. Thus, ADSCs are regarded as ideal seed cells for cartilage tissue engineering (Yoshizato et al., 2026; Zuk et al., 2002; Ong et al., 2021). In the present study, rat ADSCs were successfully isolated and cultured via collagenase I digestion combined with adherent culture. Flow cytometry results demonstrated that passage 3 (P3) ADSCs highly expressed MSC surface markers including CD29, CD44 and CD105, while barely expressed endothelial and hematopoietic cell markers CD31 and CD45. These phenotypes conformed to the international identification criteria for MSCs, confirming that the isolated cells had high purity, stable phenotypes and no mixed cell contamination, which laid a solid cellular foundation for subsequent 3D culture and chondrogenic induction. Morphological observation showed that passaged ADSCs maintained stable growth and robust proliferative activity and could be stably subcultured, further verifying the feasibility and superiority of ADSCs as seed cells.

The biocompatibility and 3D structural characteristics of scaffolds directly affect the adhesion, proliferation and differentiation of seed cells. An optimal scaffold for cartilage tissue engineering should possess favorable biosafety, appropriate spatial structure and controllable degradability. As a natural polysaccharide biomaterial, NaAlg can form 3D porous hydrogel scaffolds via calcium ion crosslinking. It can encapsulate seed cells gently, maintain the spherical physiological morphology of cells and simulate the 3D microenvironment of *in vivo* cartilage growth, and has been widely applied in cartilage regeneration research (Rothrauff et al., 2018; Iseki et al., 2024; Liu et al., 2022b). Multiple assays including live/dead cell staining, cytoskeleton staining, scratch wound assay, Transwell migration assay and CCK-8 assay were performed to evaluate the biocompatibility of sodium alginate hydrogel. The results revealed that the cell morphology, cytoskeletal structure, migration ability and proliferative activity of ADSCs in the NaAlg hydrogel scaffold co-culture system showed no significant differences compared with the control group, and cell viability remained at a high level. These findings indicated that the NaAlg hydrogel scaffold exhibited negligible cytotoxicity and did not disrupt the normal physiological functions of ADSCs. The scaffold possesses favorable biosafety and cell compatibility, serving as a promising carrier for the *in vitro* 3D culture of ADSCs, which outperforms conventional two-dimensional (2D) culture systems. Given the aforementioned properties, alginate-based hydrogels represent an invaluable 3D cell culture platform for tissue engineering (Zhang et al., 2026; Wang et al., 2021; Taira et al., 2018; Levi et al., 2022). A comparative study by Wang et al. also confirmed that multiple performance indicators of the 3D alginate hydrogel culture system were significantly superior to the conventional 2D monolayer culture system for human pluripotent stem cell-derived endothelial cells (Wang et al., 2021). Nevertheless, previous studies (Liu et al., 2022a; Abraham et al., 2025; Shukla et al., 2025; Liu and Ding, 2025) have reported that pure NaAlg hydrogel scaffolds exhibit weak cell adhesion and insufficient chondroinductive capacity. Relying merely on the physical support of scaffolds cannot efficiently facilitate the directed chondrogenic differentiation of stem cells, thereby necessitating the combined application of specific inductive factors.

Cartilage-derived morphogenetic protein-1 (CDMP-1), also known as growth differentiation factor-5 (GDF-5) or bone morphogenetic protein-14 (BMP-14), is an important member of the transforming growth factor-β (TGF-β) superfamily. It acts as a core inducer that regulates cartilage development and directed chondrogenic differentiation of stem cells. CDMP-1 can specifically activate chondrogenic signaling pathways, upregulate the expression of key chondrogenic transcription factors and matrix proteins, and inhibit hypertrophic degeneration of chondrocytes, thus playing a pivotal regulatory role in cartilage regeneration and repair (Mang et al., 2020; Tsumaki et al., 1999; Qu et al., 2018). Our results confirmed that exogenous supplementation of CDMP-1 markedly promoted the proliferation and directed chondrogenic differentiation of ADSCs. CCK-8 assays showed that the proliferative activity of ADSCs in the CDMP-1 treatment group was significantly higher than that in the pure cell group and the scaffold-only group. Moreover, the combination of CDMP-1 and alginate 3D culture system exerted the strongest pro-proliferative effect, suggesting that CDMP-1 effectively activated the proliferative potential of ADSCs, and the 3D scaffold microenvironment further amplified its biological effects. However, live/dead staining demonstrated significant differences between CDMP-1-treated groups and non-CDMP-1-treated groups, while no statistical differences existed between NaAlg-treated groups and non-NaAlg-treated groups. These results suggest that CDMP-1 facilitates the proliferation and chondrogenic differentiation of ADSCs, whereas the scaffold material exerts a limited influence on cell proliferation. Such observations can be attributed to the combined effects of multiple factors. NaAlg possesses relatively high biological inertness and insufficient cell-binding motifs. However, its excellent hydrophilicity (water retention rate of 74%–94%) enables effective simulation of the native tissue microenvironment, supporting efficient nutrient diffusion, signal molecule delivery and oxygen permeation, which is conducive to sustaining cell viability and proliferation (Ren et al., 2024; Barros da Silva et al., 2024; Liu et al., 2021b; Singh and Pramanik, 2023; Hernández-González et al., 2020; Zheng et al., 2023). In addition, CDMP-1 has been reported to promote the proliferation of mesenchymal stem cells.

Chondrogenic marker staining and molecular biological assays further validated the synergistic chondrogenic effects of CDMP-1 and NaAlg hydrogel scaffolds. Toluidine blue staining and COL-II immunohistochemical staining revealed that chondrocyte-specific proteoglycans and COL-II were secreted in all induction groups, and their expression levels increased significantly with prolonged induction time, confirming the successful directed chondrogenic differentiation of ADSCs. Notably, the deposition of cartilage matrix in the CDMP-1 combined NaAlg hydrogel scaffold group was remarkably higher than that in the CDMP-1 2D induction group and the scaffold-only group, demonstrating a synergistic effect between the 3D culture microenvironment and CDMP-1. Immunofluorescence and qRT-PCR results indicated that CDMP-1 significantly upregulated the mRNA and protein expression of core chondrogenic markers SOX9, COL-II and ACAN, and such pro-differentiation effects were further enhanced under 3D culture conditions. The underlying mechanism may be explained as follows: the 3D structure of NaAlg hydrogel scaffolds mimics the growth pattern of chondrocytes *in vivo*, maintains the stemness and differentiation activity of stem cells, and provides a stable microenvironment for CDMP-1 to exert its inductive function. In turn, CDMP-1 activates the core SOX9-mediated chondrogenic signaling pathway to promote the synthesis of cartilage-specific matrix, compensating for the lack of biological inductive activity of pure NaAlg hydrogel scaffolds. Collectively, the two components form a combined system integrating physical support and biological induction for cartilage repair.

In addition, no significant differences were observed in the expression of chondrogenic genes and proteins between the pure 3D alginate group and the 2D cell-only group, indicating that the scaffold alone only provided physical support without intrinsic chondroinductive ability. In contrast, CDMP-1 treatment substantially improved the chondrogenic differentiation capacity of ADSCs, and the advantages of the 3D composite system became more prominent with extended culture duration. These results fully verified that CDMP-1 serves as the core driver for the chondrogenic differentiation of ADSCs, while the NaAlg hydrogel scaffold acts as an optimal carrier to remodel the differentiation microenvironment. The combination of the two achieves complementary advantages and efficiently promotes the *in vitro* chondrogenic differentiation of ADSCs.

Several limitations exist in this study. First, only *in vitro* cellular experiments were conducted, and the *in vivo* chondrogenic repair efficacy and biocompatibility of the composite scaffold remain to be verified. Second, the specific molecular mechanisms and upstream/downstream signaling pathways by which CDMP-1 modulates ADSC chondrogenesis were not explored in depth. Third, systematic characterization of the mechanical properties and *in vivo* degradation rate of the composite scaffold was not performed. Further studies will focus on establishing animal models of cartilage defects to conduct *in vivo* repair experiments, elucidate the detailed molecular regulatory mechanisms, optimize the scaffold formulation and CDMP-1 working concentration. The above efforts will provide more comprehensive theoretical and experimental basis for the clinical translation of this composite system.

## 5 Conclusion

This study systematically evaluated the biosafety of NaAlg hydrogel scaffolds and the chondrogenic differentiation of ADSCs seeded on composite scaffolds induced by CDMP-1 through a series of *in vitro* experiments. The results demonstrated that the isolated rat ADSCs exhibited favorable biological characteristics, which made them ideal seed cells for cartilage tissue engineering. NaAlg hydrogel scaffolds possessed excellent biocompatibility and biosafety, serving as a promising carrier for the 3D culture of ADSCs. CDMP-1 could facilitate the proliferation and directed chondrogenic differentiation of ADSCs, and markedly upregulate the expression of chondrogenic markers including SOX9, COL-II and ACAN. Additionally, a prominent synergistic effect was observed between CDMP-1 and NaAlg hydrogel scaffolds. The 3D culture microenvironment further enhanced the chondrogenic induction of CDMP-1. Compared with conventional 2D culture and single intervention approaches, the composite system of CDMP-1 combined with ADSCs/sodium alginate hydrogel achieved the optimal chondrogenic differentiation efficacy. This novel tissue-engineered cartilage repair system integrating physical support and biological induction provides a new strategy for the efficient repair of cartilage defects, and holds great research value and promising clinical application prospects.

## Data Availability

The original contributions presented in the study are included in the article/supplementary material, further inquiries can be directed to the corresponding authors.
